# Five cases of skin desquamation due to bevacizumab combined with docetaxel-carboplatin in ovarian cancer

**DOI:** 10.1007/s13691-022-00576-5

**Published:** 2022-09-25

**Authors:** Megumi Tokunaga, Shuichi Nawata, Takayuki Komoto, Rei Mathuura, Daisuke Ichikura, Toru Watanabe, Tadanori Sasaki

**Affiliations:** 1 Department of Hospital Pharmaceutics, Showa University School of Pharmaceutical Sciences, Tokyo, Japan; 2 Department of Pharmacy, Showa University Northern Yokohama Hospital, Kanagawa, Japan; 3 Department of Pharmacy, Showa University Hospital, Tokyo, Japan; 4 Obstetrics and Gynecology, Showa University Hospital, Tokyo, Japan; 5 Obstetrics and Gynecology, Showa University Northern Yokohama Hospital, Kanagawa, Japan; 6 General Pharmacy Department, Showa University School of Pharmaceutical Sciences, Tokyo, Japan

**Keywords:** Ovarian cancer, Bevacizumab, Docetaxel-carboplatin, Skin desquamation

## Abstract

The standard of care for ovarian cancer chemotherapy is paclitaxel-carboplatin. In Stage III and Stage IV patients, the addition of bevacizumab has been reported to be effective, and bevacizumab combined with paclitaxel-carboplatin and bevacizumab combined with docetaxel-carboplatin are used. Patients who received bevacizumab combined with docetaxel-carboplatin experienced a high incidence of skin hardening followed by peeling. In patients treated with bevacizumab combined with docetaxel-carboplatin, we experienced a high incidence of post-sclerotic peeling of the skin, a symptom that is rarely seen with paclitaxel-carboplatin (TC), docetaxel-carboplatin (DC), or bevacizumab combined with paclitaxel-carboplatin, and has been reported in a few cases. Therefore, we investigated the actual situation of skin desquamation caused by bevacizumab combined with docetaxel-carboplatin. Thirty-one patients were included in the study, and their age (mean ± SD) was 62.9 ± 9.0. The breakdown of treatment was as follows: TC in nine patients, bevacizumab combined with paclitaxel-carboplatin in ten patients, DC in six patients, and bevacizumab combined with docetaxel-carboplatin in six patients. No number of patients with TC or bevacizumab combined with paclitaxel-carboplatin showed skin desquamation. One for DC, and five for bevacizumab combined with docetaxel-carboplatin. The five patients treated with bevacizumab combined with docetaxel-carboplatin improved with topical steroids and moisturizers, but symptoms repeatedly appeared after each course. Skin desquamation was more frequent in bevacizumab combined with docetaxel-carboplatin.

## Introduction

The standard for ovarian cancer chemotherapy is paclitaxel-carboplatin (TC) [1, 2]. However, some patients cannot use paclitaxel (PTX) due to allergic symptoms. PTX is a drug that frequently causes peripheral neuropathy. It correlates with the single-dose and total dose. At a median dose of 1100 mg/m^2^, 76.8% of patients in all grades and 5.0% in grade 3 or higher reported sensory deprivation symptoms. Neuropathic pain may also occur. In advanced cases, autonomic symptoms such as burning sensation with distal extremity predominance, sensory disturbance involving all senses, sensory-motor disturbance, and bradyarrhythmia may also occur [3]. Therefore, there are cases in which continuous PTX administration is complicated. Docetaxel (DTX) can be used without alcohol, reducing allergy risk. It is also reported to carry less risk of peripheral neuropathy than PTX [3]. In 2004, a study (SCOTROC1) comparing docetaxel-carboplatin (DC) and TC reported that there was no difference in response rate and Progression-Free Survival (PFS) between the two therapies [4]. Since this report, DC has been considered for patients who have difficulty using PTX.

Bevacizumab (BV) is an antibody–drug against vascular endothelial growth factors. In the GOG218 study [5], a representative clinical trial, bevacizumab combined with docetaxel-carboplatin with a change to DTX was allowed in patients who had difficulty using PTX due to allergy onset or peripheral neuropathy of Grade three or higher [5]. However, there is no information on the number of patients converted to bevacizumab combined with docetaxel-carboplatin or adverse events, and there are few reports on actual clinical practice. At Showa University Northern Yokohama Hospital, we experienced a high frequency of symptoms of peeling after skin hardening in patients treated with bevacizumab combined with docetaxel-carboplatin. This symptom has not been reported with TC or bevacizumab combined with paclitaxel-carboplatin [5]. It has also not been reported in DC [4]. Therefore, we will discuss the clinical picture of the appearance of dermabrasion and report it with a literature discussion.

## Methods.

Thirty-one patients who underwent TC, bevacizumab combined with paclitaxel-carboplatin, DC, or bevacizumab combined with docetaxel-carboplatin for ovarian cancer at our institution from January 2019 to December 2019 were included in the study. The evaluation item was the presence or absence of dermabrasion, and the medical records were examined retrospectively. Ethical approval for the present study was obtained from the local ethics committee (approval no: 19H084).

## Results

The age (mean ± SD) of the 31 patients was 62.9 ± 9.0. Postoperative adjuvant therapy was used in 23 patients, unsuitable for surgery in three patients and recurrence in five patients. The breakdown of regimens was TC in nine patients, bevacizumab combined with paclitaxel-carboplatin in ten patients, DC in six patients, and bevacizumab combined with docetaxel-carboplatin in six patients. The regimens included TC in nine, bevacizumab combined with paclitaxel-carboplatin in ten, DC in six, and bevacizumab combined with docetaxel-carboplatin in six. The number of patients with skin desquamation was zero for TC, 0 for bevacizumab combined with paclitaxel-carboplatin, one for DC, and five for bevacizumab combined with docetaxel-carboplatin. A summary of the five six cases that presented with dermabrasion is shown in Table 1. Swelling and edema appeared first, followed by skin hardening and then peeling. Symptoms were alleviated with topical steroids and moisturizers but flared up after each course. In most cases, the symptoms improved markedly after a change in regimen or completion of chemotherapy.

**Table 1:** Case file

Case	Stage of disease	Regimen	Dose	Symptom onset	Symptom location /characteristics	Grade^b^	Medicine	Outcome	History of allergy
1	Progressive recurrence Stage IVB	DC+BV^a^	100%	4 Courses	Cheek, sole redness + pain +	G2	Clobetasol 0.05% white petrolatum	Significant improvement after regimen change	Milk
2	Adjuvant chemotherapy Stage IVB	DC+BV^a^	100%	1 Course	Sole, heel redness ± pain +	G2	Betamethasone 0.05% urea 10%	Significant improvement after regimen change	Alcohol
3	Adjuvant chemotherapy Stage IIIB	DC+BV^a^	100%	1 Course	Palm, heel redness − pain ±	G2	Betamethasone 0.05% white petrolatum	Significant improvement after regimen change	None
4	Progressive recurrence Stage IVB	DC+BV^a^	80%	1 Course	Arm, palm redness − pain ±	G2	Clobetasol 0.05% heparinoid	Significant improvement after regimen change	Cat tapes latex
5	Adjuvant chemotherapy Stage IVB	DC+BV^a^	100%	1 Course	Palm redness − pain ±	G2	Betamethasone 0.05% white petrolatum	Gradual improvement after regimen change	Alcohol

Details are given on 5 cases of skin desquamation in DC+BV. Case 1.4 was inoperable and progressive. Case 2.3.5 was treated with anticancer agents as adjuvant chemotherapy after surgery. Case 1 developed skin desquamation in after the fourth course, and the others after the first course. Most cases experienced redness and pain on the palms and soles, degree G2. Symptoms improved with topical steroids and moisturizers. In all cases, symptoms improved after the regimen change

^a^DC+BV: bevacizumab combined with carboplatin-docetaxel

^b^The grading system is based on CTCAE v5.0.

**Fig. 1:**
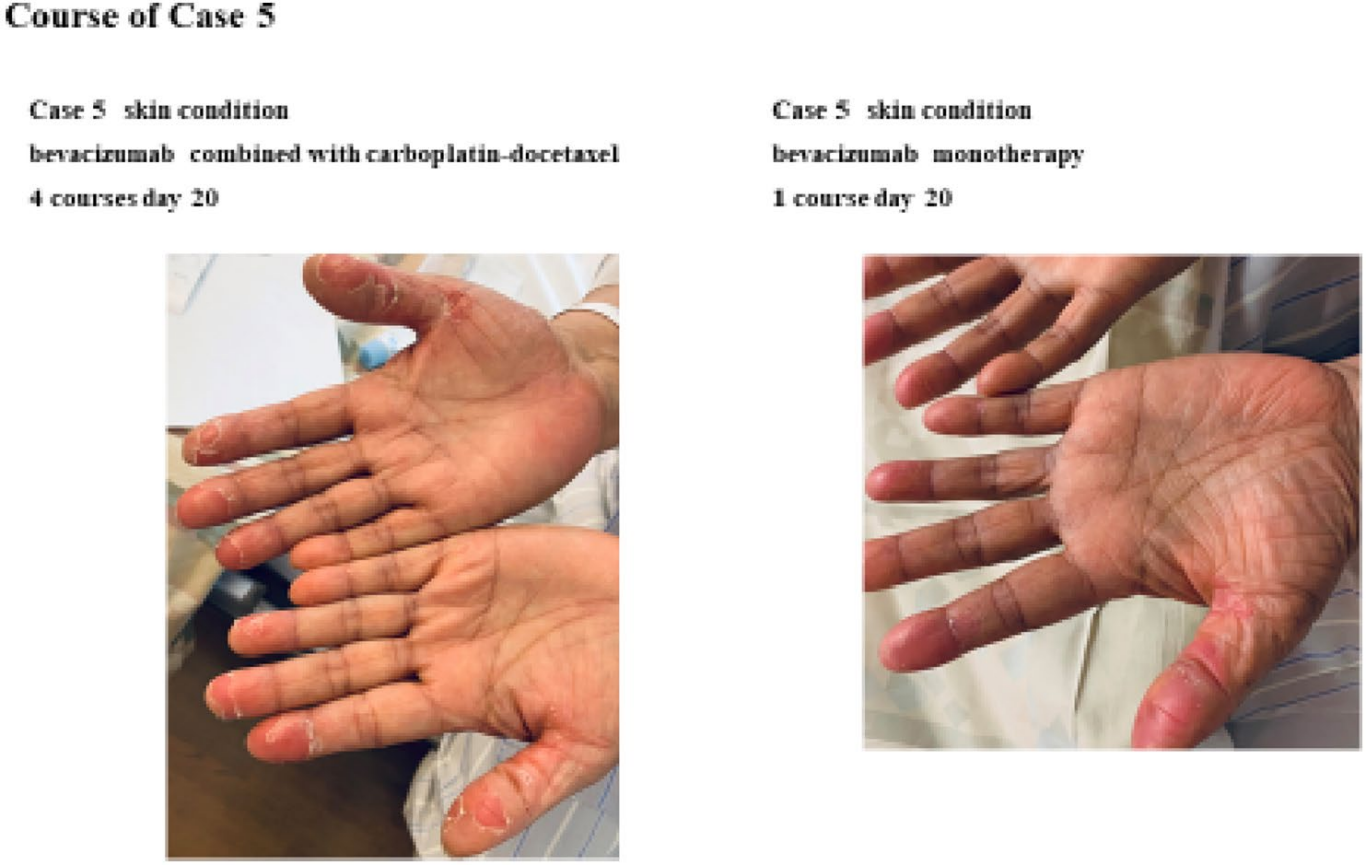
Course of case file

In Table 1, a summary of each case is presented. Five cases of skin desquamation caused by BV combined with DC are described in detail. Case 1.4 was of untreatable and progressive cancer. After the surgery, Case 2.3.5 received chemotherapy that included anticancer drugs as adjuvant therapy. After the fourth course of treatment, Case one experienced skin desquamation, whereas the other cases experienced skin desquamation after the first course. Most cases had degree G2 redness and pain on the palms and soles. Topical steroids and moisturizers were used to reduce symptoms in these cases. After changing the regimen, symptoms improved in all cases. The details of Case five, a representative case, are shown below, and a photograph of the course of skin desquamation is shown in Fig. 1.

### Case 5

Sixty-five years old, female. Stage IVB ovarian cancer. History of bilateral hip osteoarthritis. History of allergy to alcohol. After four courses of DC as preoperative chemotherapy, the patient underwent tumor reduction surgery. After surgery, bevacizumab combined with docetaxel-carboplatin was administered, and after one course, intense skin desquamation occurred on the palms. The swelling was seen, and the skin hardened and then peeled. The pain was mild, with no redness. After four courses of bevacizumab combined with docetaxel-carboplatin, the symptoms improved with the switch to BV monotherapy.

## Discussion

Bevacizumab combined with docetaxel-carboplatin was associated with a higher frequency of skin desquamation than TC, bevacizumab combined with paclitaxel-carboplatin, or DC.

BV binds explicitly to human vascular endothelial growth factor (VEGF), thereby inhibiting the binding of VEGF to the VEGF receptor expressed on vascular endothelial cells and blocking the VEGF signaling pathway. The blockade of VEGF signaling inhibits angiogenesis in tumor tissue, which VEGF plays a role in, and thus inhibits tumor growth. The package insert for Avastin 2005 for intravenous infusion states that skin desquamation of BV is less than 1%. When used in combination with chemotherapy, skin disorders such as exfoliative dermatitis and rash have been reported to occur in 19–46% of patients and delayed wound healing in 13% of patients [6, 7]. All of the skin disorders in this report resolved spontaneously after treatment was completed [7]. This is similar to the course of the present case. DTX inhibits the depolymerization of microtubules, which play an essential role in cell division, and causes stabilization and hyperplasia of microtubules, thereby inhibiting cell division and producing an antitumor effect. As for adverse skin reactions, hypersensitivity reactions such as urticaria and edematous dermatitis are common, but there are a few reports of scleroderma-like symptoms and exfoliative symptoms caused by DTX. Cleveland et al. reported a patient with extensive edema and subsequent exfoliation after DTX in metastatic breast cancer [8], and Hasset et al. also reported scleroderma-like symptoms [9]. In Japan, Itoh et al. reported five cases of edema of the extremities and subsequent hardening of the skin after administration of taxanes. All five patients had a history of DTX use [10]. Although the total dose of DTX in the five patients varied, it was argued that the edema was dose-dependent and that the skin hardening was also likely to be dose-dependent [10]. Of the five cases we experienced in this study, four presented with skin exfoliation symptoms at the beginning, which was different from the onset of skin symptoms caused by DTX in the past. After the onset of symptoms, topical steroids were used for treatment, and the patients improved quickly without worsening. It has been reported that the use of steroids was influential in the treatment of skin symptoms caused by DTX in the past [8–10]. Although the onset of symptoms was different, steroids were also thought to be effective in this condition. However, bevacizumab combined with docetaxel-carboplatin tends to repeat relapse after each cool but not worsen. Furthermore, DTX and BV could be used without weight loss. Although the procedure was performed safely in this case, skin desquamation may be induced by an allergic reaction. As a result, after the occurrence of skin desquamation, extreme caution should be used while administering bevacizumab combined with docetaxel-carboplatin.

We reported the characteristic skin desquamation of bevacizumab combined with docetaxel-carboplatin. The mechanism by which bevacizumab combined with docetaxel-carboplatin causes a high frequency of skin peeling is unknown, but it is suggested that the skin symptoms of DTX may be a synergistic manifestation of the dermatitis of BV. Vascular and endothelial cadherin repeats are relaxed by VEGF, which also controls vascular permeability [11]. This adverse effect may be the cause of skin desquamation that occurs when DTX and BV are combined. However, this report may provide helpful information for the effective and safe implementation of bevacizumab combined with docetaxel-carboplatin.
